# Takotsubo syndrome and cardiac implantable electronic device therapy

**DOI:** 10.1038/s41598-019-52929-5

**Published:** 2019-11-12

**Authors:** Ibrahim El-Battrawy, Julia W. Erath, Siegfried Lang, Uzair Ansari, Michael Behnes, Thorsten Gietzen, Xiaobo Zhou, Martin Borggrefe, Ibrahim Akin

**Affiliations:** 1 First Department of Medicine, Faculty of Medicine, University Medical Centre Mannheim (UMM), University of Heidelberg, Mannheim, Germany; 2 DZHK (GermanCenter for Cardiovascular Research), Partner Site, Heidelberg-Mannheim, Mannheim, Germany; 3 Department of Cardiology/Division of Clinical Electrophysiology, University Hospital Frankfurt, Goethe University, Frankfurt a. M., Germany

**Keywords:** Cardiac device therapy, Interventional cardiology

## Abstract

Recent studies have reported that takotsubo syndrome (TTS) patients are suffering from life-threatening arrhythmias. The aim of our study was to understand the short and long-term usefulness of cardiac implantable electronic devices in TTS patients.We constituted a collective of 142 patients in a bi-centric study diagnosed with TTS between 2003 and 2017. The patient groups, divided according to the treatment with (n = 9, 6.3%) or without cardiac devices (n = 133, 93.7%), were followed-up to determine the importance of devices and its complications. One patient was treated with a permanent pacemaker, five patients with a wearable cardioverter defibrillator, two patients with a subcutaneous defibrillator and one patient with a transvenous defibrillator. Regular device check-up was documented in all patients, presenting an ongoing high-degree AV-block. Neither device complications nor life-threatening tachyarrhythmias were documented after acute TTS event. However, patients comprising the device group suffered significantly more often from a highly reduced EF (30 ± 7.7% versus 39.1 ± 9.7%; p < 0.05), cardiogenic shock with use of inotropic agents (66.6% versus 16.6%; p < 0.05) and cardiopulmonary resuscitation (44.4% versus 5.3%; p < 0.05). Our data confirm the usefulness of pacemaker in TTS patients. However, the cardioverter defibrillator including wearable cardioverter defibrillator may not be recommended.

## Introduction

Takotsubo syndrome (TTS) is characterized by an acute heart failure syndrome and associated with a bevy of adverse events^1–5^. Four forms of TTS according to affected wall motion changes have been described^1,3,6–8^. Despite the reversibility of TTS recently published data have described a comparable outcome with ACS^3,9^. The declined outcome is reflected by TTS associated complications including arrhythmias. It has been debated that life-threatening arrhythmias are common in up to 14% of TTS cases and related to QTc interval prolongation^10–12^.The management of arrhythmias in TTS challenges physicians based on the reversible character.

The present study aimed to investigate the role of cardiac implantable electronic devices (CiED) in TTS syndrome and long-term follow-up of its importance and associated complications.

## Methods

We recruited 142 patients diagnosed with TTS between January 2003 and September 2017 at the Medical Faculty of Mannheim and University of Frankfurt. The diagnosis TTS was reviewed by two independent experienced cardiologists on the basis of the Mayo Clinic Criteria^13,14^.

The study protocol received ethics approval from the Ethics Committee of the Medical Faculty Mannheim, University of Heidelberg^4^. The need for informed consent was waived by the ethics committee. All methods were performed in accordance with the relevant guidelines and regulations.

Cases were divided into two groups according the use of CiED including transient and permanent pacemaker, transvenous ICD, subcutaneous ICD and wearable cardioverter defibrillator or not. CiED was recommended due to the occurrence of life-threatening arrhythmias (encompassing ventricular tachycardia, ventricular fibrillation as well as complete atrioventricular block) and the persistence of reduced ejection fraction (EF) at discharge, all of which were assessed by ECG and echocardiography independently reviewed by two experienced cardiologists prior admission, at admission or during hospital stay. Evaluation the rate of recurrence of life-threatening arrhythmias at short- and long-term was conducted using chart review and medical records.

Different TTS related complications and the outcome of patients including arrhythmias with use of device therapy, thromboembolic events, pulmonary congestion with the need for ventilation, cardiogenic shock and subsequent use of inotropic agents were assessed at TTS event and over follow-up by chart-review and telephone review. The cause of death was evaluated using the above mentioned assessments. Unknown cause of death was defined in cases with unclear cause of death.

### Statistics

SPSS statistics 23.0 was used for statistical analysis. Continuous variables with normal distribution and non-continuous variables not normal normal distribution were presented as means ± standard deviation or median with interquartile range, which have been compared using student’s t-test and the Mann–Whitney U-test. A frequency (%) was used for categorical variables and compared using Chi-squared-test or Fisher’s exact test. Statistical significance was defined as a p value ≤ 0.05.

## Results

### Baseline demographics

We studied the clinical and echocardiographic data of 142 TTS patients. Of these, in 9 (6.3%) patients device implantation was undertaken. The indication for CiED implantation was as following; ventricular fibrillation (n = mp2), torsade de pointes (n = 2), completely atrioventricular-Block (n = 1), and highly reduced EF at discharge (n = 4). The baseline characteristics of patients, who received a CiED are illustrated in Table 1.

**Table 1:** Baseline characteristics of 142 patients initially presenting with TTS.

Variables	*No Device* (n = 132)	*Device* (n = 9)	p value*
Demographics			
Age, mean ± SD	66.5 ± 11.1	59.5 ± 26.0	0.43
Female, n (%)	112 (85)	8 (89)	0.74
Symptoms, n (%)			
Dyspnea	52 (39)	5 (55.5)	0.48
Chest pain	67 (50.7)	3 (33.3)	0.71
Clinic parameter			
Systolic BP, mmHg	133.3 ± 30.2	117 ± 50	0.38
Diastolic BP, mmHg	78.3 ± 15.8	67.4 ± 26.8	0.34
Heart rate, bpm	99.6 ± 25.7	84.3 ± 24.5	0.08
ECG Data, n (%)			
ST-segment elevation	40 (30)	1 (11.1)	0.67
Inversed T-Waves	117 (88)	8 (89)	0.65
PQ-interval	159 ± 8	161.7 ± 26	0.82
QTc (ms), mean ± SD	474.3 ± 62.2	454.4 ± 57.4	0.34
Stress factor, n (%)			
Emotional sress	40 (30)	3 (33.3)	1.00
Physical stress	62 (47)	3 (33.3)	0.50
Laboratory values, mean ± SD			
C-Reactive protein (mg/l)	49.4 ± 77.8	14.9 ± 29	0.01
Hemoglobin	12.2 ± 2.0	12.0 ± 2.3	0.72
Creatinine (mg/dl)	1.1 ± 0.6	11.2 ± 29.2	0.33
Echocardiography data, n (%)			
LV EF%	39.1 ± 9.7	30 ± 7.7	<0.01
Follow-up LV EF %	52.5 ± 10.6	43.3 ± 17.3	0.15
Apical ballooning	94 (71.2)	8 (89)	0.26
Mitral regurgation	62 (47)	6 (66.6)	0.20
Tricuspid regurgation	49 (37)	6 (66.6)	0.08
RV-Involvement	28 (21.2)	2 (22.2)	1.00
Medical history, n (%)			
Smoking	41 (31)	1 (11.1)	0.28
Diabetes mellitus	28 (21.2)	3 (33.3)	0.45
BMI MI25 kg/m²	33 (25)	6 (66.6)	0.02
Hypertension	78 (59)	5 (55.5)	1.00
COPD	27 (20)	0 (0)	0.20
Atrial fibrillation	23 (17.4)	2 (22.2)	0.67
Coronary artery disease	22 (16.6)	2 (22.2)	1.00
History of malignancy	15 (11.3)	2 (22.2)	0.29
Drugs on admission, n (%)			
Beta-blocker	44 (33.3)	4 (44.4)	0.46
ACE inhibitor	50 (38)	3 (33.3)	1.00
ARB	9 (7)	3 (33.3)	0.03
Aspirin	34 (26)	3 (33.3)	0.68

Baseline characteristics of patients presenting with TTS according to implantation of devices. *p values for the comparison between *no device implantation and device implantation*; SD, Standard deviation; ECG, Electrocardiogram; EF, Ejection fraction; BMI, body-mass-index, COPD, Chronic obstructive pulmonary disease; ACE, Angiotensin-convetring-enzyme; ARB, Angiotesin-receptor-blocker.

### In-hospital complications

Patients treated with CiED suffered more frequently of in-hospital complications; Table 2. Cardiogenic shock was more frequently documented in the CiED group (66.6%) as compared to the non-CiED group (16.6%); p < 0.01. Consequently, the use of positive inotropic drugs was significantly higher in the device group (55.5% versus 16%; p = 0.01). Cardiopulmonary resuscitation (44.4% versus 5.5%; p < 0.01) was significantly more presented in the device group. Patients treated with CiED tended to stay longer in the hospital than patients without CiED treatment (8.3 ± 6.0 versus 4.5 ± 6.0 days; p = 0.07). Other TTS related complications including respiratory support and intubation were similarly documented in both groups.

**Table 2:** Events and treatment strategy.

Variables	*No device* (n = 132)	*Device* (n = 9)	p value*
NPPV and intubation	78 (59)	4 (44.4)	0.49
Inotropic agents	21 (16)	5 (55.5)	0.01
Resuscitation	7 (5.3)	4 (44.4)	(0.01
VA-ECMO	1 (0.75)	0 (0)	1.00
Admission to ICU, length of stay	4.5 ± 6.0	8.3 ± 6.0	0.07
In-hospital death	10 (7.5)	0 (0)	1.00
Acquired Long QTs	85 (64.4)	5 (55.5)	0.47
Cardiogenic shock	22 (16.6)	6 (66.6)	<0.01
Malignant arrhythmia	9 (7)	4 (44.4)	<0.01

In-hospital events in TTS patients with and without device implantation. *p values for the comparison between *no device implantation and device implantation*; NPPV, Noninvasive positive pressure ventilation; VA-ECMO, Veno-arterial extracorporal membrane oxygenation; ICU, Intermediate care unit.

### CiED implantations and follow-up

#### Pacemaker

One TTS patient presented an apical form. The same patient was admitted to the hospital two years later due to recurrent syncope. Echocardiography and coronary angiography presented a recurrent TTS. This patient received a temporary pacemaker at admission. Due to persistent bradycardia and atrioventricular block III, a permanent pacemaker implantation was done. Regular device check-ups over 8 years presented ongoing bradycardia and AV-block III. No complications have been documented.

#### Transvenous ICD

**Figure 1:**
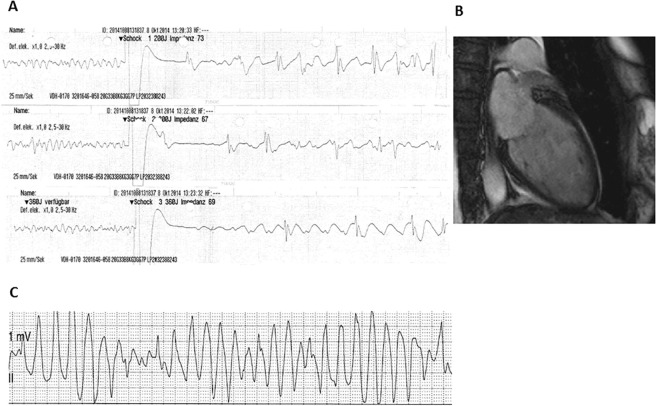
(**A**) Cumulative ECG shows recurrent ventricular fibrillation in a female patient with recurrent use of cardioverter defibrillator. (**B**) Cardiac MRI shows a midventricular TTS. The patient has been resuscitated and defibrillated 8 times. (**C**) ECG of a TTS patient presents torsade de pointes.

One patient was admitted from a referral hospital due to bradycardia and hypoglycemia. One day after admission torsade de pointes has been documented and terminated with a cardioversion (Fig. 1C). A coronary angiography excluded relevant stenosis. However, echocardiography and laevocardiography presented a biventricular TTS form of apical form. CiED implantation was undertaken. Ongoing regular device-follow-up showed no more bradycardia or life-threatening arrhythmia. Additionally no complications have been documented over the follow-up.

#### Subcutaneous ICD

Subcutaneous ICD (s-ICD) has been implanted in two patients. The first patient was admitted to the hospital for a coloscopy. During coloscopy cardiopulmonary resuscitation because of ventricular fibrillation was required (Fig. 1A). Cardiac MRI, coronary angiography and echocardiography showed a midventricular TTS. Due to recurrent ventricular fibrillation defibrillation was performed eight times. Because of the recurrent ventricular fibrillation ICD implantation was undertaken. During the follow-up of 2 years no complications or life-threatening arrhythmias have been documented.

The second patient was a 22 years old female. The patient was admitted to the hospital due ventricular fibrillation. ECG documentation presented a long QT syndrome. Additionally, an old ECG recorded 4 years ago documented a prolonged QTc interval. There was no family history of sudden cardiac death. In-hospital diagnostics showed a highly reduced EF. Cardiac MRI confirmed an apical TTS form. A recovery of EF has been documented in the hospital. This patient underwent subcutaneous ICD implantation due to a suspected congenital long QT syndrome. Neither complications nor arrhythmias have been documented over 3 months of follow-up.

#### Wearable cardioverter defibrillator

A wearable cardioverter defibrillator was used in five TTS patients due to a highly reduced left ventricular EF. Almost three months later a recovery of EF was documented. However, one female patient (17 year old) underwent an s-ICD implantation due to a suspected congenital long QT-syndrome. No complications or life-threatening arrhythmias have been documented during the follow-up.

Table 3 illustrates a detailed follow-up of TTS treated with CiED.

**Table 3:** Device follow-up.

Sex, age	Device indication	Type of Device	Follow-up of device (days)	Complications	arrhythmia or bradycardia
Female, 55	Recurrent VF	s-ICD	1095	0	0
Female, 86	AV-Block III	Single chamber	2920	0	1
Female, 76	TDP and bradycardia	Dual chamber ICD	930	0	1
Male, 76	Highly reduced EF	WCD	84	0	0
Female, 67	Highly reduced EF	WCD	84	0	0
Female, 22	TDP and VF	s-ICD	30	0	0
Female, 51	Highly reduced EF	WCD	730	0	0
Female, 86	Highly reduced EF	WCD	365	0	0
Female, 17	VF	WCD	730	0	0

Detailed description of TTS patients underwent device therapy. Regular follow-up including complications is illustrated. s-ICD: subcutaneous ICD; TDP: torsade de pointes; EF: ejection fraction; VF: ventricular fibrillation, WCD: wearable life vest.

## Discussion

Using our retrospective clinical investigation of 142 TTS patients, we are able to conclude that: (i) the use of CiED in TTS might be safe; (ii) the long-term need for pacemaker in TTS is higher as compared to ICD; (iii) due to the recovery of EF a wearable cardioverter defibrillator might be not required in TTS patients

TTS has been thought to be associated with a favorable prognosis. Recently published data have shown a similar outcome of TTS and ACS. TTS related complications including arrhythmias and acute heart failure have been reported^10,11,15^. Up to 14% of TTS patients may suffered from lifethreatening arrhythmias and triggered by a QTc interval prolongation. The management of such complication remains challenging physicians due to the reversible character of TTS and absence of risk stratification strategies.

Our study is the largest, to the best of our knowledge, which describes the use of permanent and temporary CiED in TTS.

Stiermaier *et al*. has described a higher in-hospital and one-year mortality rate of TTS patients associated with life-threatening arrhythmias including ventricular fibrillation, ventricular tachycardia and complete atrioventricular block^11^. It has been reported that one patient was treated by ICD implantation and one patient was discharged with a wearable cardioverter defibrillator. Follow-up did not record any relevant arrhythmias^16^. Here we report about the use of wearable cardioverter-defibrillator in 5 patients and s-ICD in 2 patients. No arrhythmias have been documented over long-term follow-up. One young patient treated with a wearable cardioverter defibrillator has undergone an s-ICD due to a congenital long QT. All these data suggest a consensus that cardioverter defibrillator including wearable cardioverter defibrillator are not recommended in TTS patients. The higher vulnerability of myocardial muscle to develop ventricular arrhythmias at TTS event might be explained by oedema^2^. Another important trigger for ventricular arrhythmias is the acquired long-QT syndrome. Long QT syndrome is associated with sudden cardiac death. Recently published data of a TTS model using induced pluripotent stem cells showed that estradiol attenuates the acquired long-QT syndrome^17^, consistent with the clinical observation that TTS occurs frequently in postmenopausal women. Of note, in our current study, we found a concomitant long QT syndrome and TTS at the same time. Both patients were young. Therefore, a congenital channelopathy should be evaluated using family screening and genetic screening.

Cardiac arrest could be in presented in TTS patients in absence of QT prolongation. The main mechanism for this finding might be related to catecholamine toxicity. In other cases TTS might be the results of cardiac arrest and cardiopulmonary resuscitation^18^.

The reversible character of wall motion abnormality of TTS may explain the uncertain value of cardioverter defibrillators (ICD and wearable life-vest) as showed in our study^19^. In contrast, our findings in one patient with permanent pacemaker showed that a permanent device might be required in TTS patients with bradycardia or atrioventricular block. Our data are comparable with the study of Stiermaier *et al*. on the use of pacemaker in TTS patients.

The ongoing susceptibility to fatal bradyarrhythmias in TTS patients after index-event and lower-susceptibility to fatal tachyarrhythmias after the acute TTS event might be explained by the recommended medication (beta-blockers) at discharge. Beta-blockers suppress ventricular tachyarrhythmias, but nervertheless decrease the heart rate and might be the cause for permanent pacing of pacemakers. We have recently presented in our TTS model that beta-blockers shorten the action potential duration consisting with a shortening of QT-interval and reducing the arrhythmia risk.

### Study limitations

This study is a bi-centric retrospective case series study with a low number of patients and without a comparative control group. Therefore the non-significant comparison values of baseline characteristics should be evaluated with caution. Magnetic resonance imaging was not performed systematically to find a possible interaction between remnant edema and/or inflammation and life-threatening arrhythmias. The use of electrophysiology work-up to stratify the risk of ventricular tachyarrhythmias in TTS patients was not evaluated.

## Conclusions

Permanent CiED implantation might be recommended in cases of bradyarrhythmias. The use of temporary and permanent cardioverter defibrillator is not recommended in TTS patients. More data on management of life-threatening arrhythmias are necessary to provide a general recommendation.

## References

[1] Dote K, Sato H, Tateishi H, Uchida T, Ishihara M (1991). [Myocardial stunning due to simultaneous multivessel coronary spasms: a review of 5 cases]. J Cardiol.

[2] Eitel I (2011). Clinical characteristics and cardiovascular magnetic resonance findings in stress (takotsubo) cardiomyopathy. JAMA.

[3] Templin C (2015). Clinical Features and Outcomes of Takotsubo (Stress) Cardiomyopathy. N Engl J Med.

[4] El-Battrawy I (2016). Prevalence, Clinical Characteristics, and Predictors of Patients with Thromboembolic Events in Takotsubo Cardiomyopathy. Clin Med Insights Cardiol.

[5] El-Battrawy I, Borggrefe M, Akin I (2017). Atrial fibrillation as a risk factor for worse outcome in acute coronary syndrome. Int J Cardiol.

[6] Ghadri JR (2016). Differences in the Clinical Profile and Outcomes of Typical and Atypical Takotsubo Syndrome: Data From the International Takotsubo Registry. JAMA Cardiol.

[7] Suzuki K (2004). An atypical case of “Takotsubo cardiomyopathy” during alcohol withdrawal: abnormality in the transient left ventricular wall motion and a remarkable elevation in the ST segment. Intern Med.

[8] Kato K, Sakai Y, Ishibashi I, Kobayashi Y (2015). Transient focal left ventricular ballooning: a new variant of Takotsubo cardiomyopathy. Eur Heart J Cardiovasc Imaging.

[9] Redfors B (2015). Mortality in takotsubo syndrome is similar to mortality in myocardial infarction - A report from the SWEDEHEART registry. Int J Cardiol.

[10] Madias C (2011). Acquired long QT syndrome from stress cardiomyopathy is associated with ventricular arrhythmias and torsades de pointes. Heart Rhythm.

[11] Stiermaier T (2015). Prevalence and Clinical Significance of Life-Threatening Arrhythmias in Takotsubo Cardiomyopathy. J Am Coll Cardiol.

[12] Stiermaier, T. *et al*. Incidence, determinants and prognostic relevance of cardiogenic shock in patients with Takotsubo cardiomyopathy. *Eur Heart J Acute Cardiovasc Care*, 10.1177/2048872615612456 (2015). 10.1177/2048872615612456 26474843

[13] Madhavan M, Prasad A (2010). Proposed Mayo Clinic criteria for the diagnosis of Tako-Tsubo cardiomyopathy and long-term prognosis. Herz.

[14] Prasad A, Lerman A, Rihal CS (2008). Apical ballooning syndrome (Tako-Tsubo or stress cardiomyopathy): a mimic of acute myocardial infarction. Am Heart J.

[15] El-Battrawy I (2017). Incidence and Prognostic Relevance of Cardiopulmonary Failure in Takotsubo Cardiomyopathy. Sci Rep.

[16] Stiermaier, T. *et al*. Management of arrhythmias in patients with Takotsubo cardiomyopathy: Is the implantation of permanent devices necessary? *Heart Rhythm*, 10.1016/j.hrthm.2016.06.013 (2016). 10.1016/j.hrthm.2016.06.013 27298201

[17] Battrawy I (2018). Estradiol protection against toxic effects of catecholamine on electrical properties in human-induced pluripotent stem cell derived cardiomyocytes. Int J Cardiol.

[18] Ghadri JR (2018). International Expert Consensus Document on Takotsubo Syndrome (Part II): Diagnostic Workup, Outcome, and Management. Eur Heart J.

[19] Migliore F (2013). Incidence and management of life-threatening arrhythmias in Takotsubo syndrome. Int J Cardiol.

